# Attitudes to ageing mediates the relationship between perception of age-friendly city and life satisfaction among middle-aged and older people in Macao: a cross-sectional study

**DOI:** 10.1186/s12877-024-04961-y

**Published:** 2024-04-23

**Authors:** Sok Leng Che, Wai In Lei, Tan Hung, Sok Man Leong

**Affiliations:** 1 Nursing and Health Education Research Centre, Kiang Wu Nursing College of Macau, Macao, SAR China; 2 Nursing and Health Education Study Centre, Kiang Wu Nursing College of Macau, Macao, SAR China; 3 Education Department, Kiang Wu Nursing College of Macau, Macao, SAR China; 4 Research Management & Development Department, Kiang Wu Nursing College of Macau, Macao, SAR China

**Keywords:** Age-friendly city, Life satisfaction, Attitudes to ageing, Community-dwelling older adults

## Abstract

**Background:**

Societal attitudes toward ageing play a significant role in shaping one’s ageing experience, and an age-friendly environment can potentially enhance the life satisfaction of older individuals. The objective of this study is to examine the role of attitudes to ageing as mediators in the association between the perception of an age-friendly city and life satisfaction among middle-aged and older adults.

**Methods:**

Using the tools of Age-Friendly City (AFC) criteria, Attitudes to Ageing Questionnaire (AAQ) to measure psychosocial loss, psychological growth, and physical change, and Satisfaction with Life Scale (SWLS) to assess the level of life satisfaction among community-dwelling middle-aged and older people in Macao. Multiple mediation analysis was performed to test the mediation effect.

**Results:**

A total of 543 participants were included in this study. The average score of AFC was 4.25, the total scores of psychosocial loss, physical change, and psychological growth were 24.06, 29.00, and 26.94 respectively. The total score of SWLS was 24.06. There was a partial mediation of attitudes to ageing in the relationship between perception of age-friendly city and life satisfaction. The mediation effect explained 56.1% of the total effect of AFC to life satisfaction.

**Conclusion:**

The development of an age-friendly city can help improve the public’s view on ageing, and thus improve their life satisfaction. It is important for government to consider the improvement of people’s attitudes to ageing when developing policies regarding AFC.

## Introduction

Macao follows the trend of the world’s population ageing, it has become an aged society in 2020 [1]. The population aged 65 years and above has reached 15.7% of total local population in 2022 [2]. Increasing ageing population causes higher government expenditure in health and social care areas [3]. The concept of ageing-in-place emerged as a substitute for institutionalization, and has been redefined as “One’s journey to maintain independence in one’s place of residence as well as to participate in one’s community”, considering space, person, and time elements [4].

Providing an ageing-in-place environment requires substantial construction in all aspects of life, from social interactions to physical facilities. It is important to note that individuals live in a society in which social and personal factors strongly impact their satisfaction with life. From a social point of view, the World Health Organization (WHO) introduced age-friendly city (AFC) in 2007 [5]. Based on focus groups with older people, caregivers, and service providers across countries, WHO identified eight key characteristics of age-friendly cities, which are (1) community and health care, (2) transportation, (3) housing, (4) social participation, (5) outdoor spaces and buildings, (6) respect and social inclusion, (7) civic participation and employment, (8) communication and information [5]. The Macao SAR government has launched the “Pension Security Mechanism and the Ten-Year Action Plan for Services for Older People from 2016 to 2025” to respond to population ageing, and to guide the services for older people. It covers wide range of domains, including medical and social services, protection of rights, social participation, and living environment [6]. Also, the “Legal Regime Guaranteeing the Rights and Interests of Olde People” takes effect in 2018 [7]. This law aims to promote an inclusive society that provides both support and a sense of belonging and usefulness for older people. In spite of these efforts, the level of age-friendly friendliness has not yet been evaluated.

The psychological well-being of middle-aged and older people can be improved by creating an environment that is age-friendly [8–12]. Because a community that recognizes and accommodates the needs of frail older adults can provide a protective buffer that will allow these individuals to remain in their homes for a longer period of time [13]. Different domain might contribute differently to the level of life satisfaction. In Spain, the domains of outdoor spaces and buildings, transportation, and community and health care are considered essential to maintain independence and mobility in older adults [14], while housing has been found to be positively related to life satisfaction in China [15, 16]. Moreover, there are age differences regarding the domains that are associated with life satisfaction. In Hong Kong, a high level of life satisfaction was associated with the domain of community and health services for those aged 65 to 74 years, while civic participation and employment were associated with higher levels of life satisfaction for those aged 75 years and older [17].

The construction of an age-friendly city demonstrates a society’s concern about population ageing. While the government may put considerable effort into creating an age-friendly society, it is important to assess how people perceive the age-friendliness of a society. Thus, perceptions of age-friendliness can be used as a measure of one’s level of inclusion within society. Furthermore, when individuals live in the city, they are aware of the expectations regarding their own ageing, thus influencing their attitudes toward ageing. Although attitudes toward ageing are an individuals’ evaluation of themselves, the expectations and discourse regarding ageing in the society in which they live can affect individuals’ views on ageing [18, 19]. Wurm et al. [20] distinguished one’s view on ageing into age stereotypes and subjective ageing; age stereotypes refer to how people apply society’s attitudes towards ageing, whereas subjective ageing is an individual’s own experiences with ageing across their lifespan. There is an inextricable link between subjective ageing and age stereotypes, as one’s ageing experience is continually shaped by society’s attitudes toward ageing. In the view of this, the current study hypothesizes that individuals’ perceptions of age-friendliness would have a positive effect on their attitudes toward ageing.

**Fig. 1:**
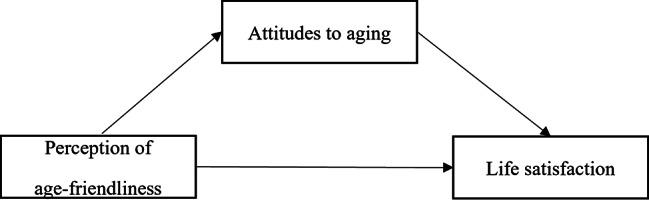
Conceptual illustration of exploratory mediation model

The impact of one’s perception of ageing on health is multidimensional [20]. Attitudes toward ageing were found to be correlated with physical and psychological health, such as heart disease, hypertension, obesity, depression, falls risk, as well as health behaviors (i.e. smoking, drinking) [21–23]. There have been extensive studies demonstrating that one’s view on ageing can have a significant long-term adverse impact on their own health [24–27]. Levy et al. [28] found that older people who hold more positive attitudes toward their ageing process lived 7.5 years longer than those who have more negative attitudes. Regarding the relationship between attitudes to ageing and life satisfaction, previous studies revealed that older people who felt younger than their chronological age presented better life satisfaction [29–31]. However, it remains unclear whether the impacts of an individual’s perception of an age-friendly city and their attitudes towards ageing on life satisfaction occur independently, or the impact of an individual’s perception of an age-friendly city is mediated by attitudes toward ageing. As of now, no pertinent research has been conducted on this matter, thus this study aims to address this research gap. The current study aims to explore the attitudes toward ageing as mediators of the relationship between the perception of age-friendly city and life satisfaction among middle-aged and older adults. It is hypothesized that individual’s perception of age-friendliness will explain individual differences in ageing attitudes and that ageing attitudes will mediate the effect of individual’s perception of age-friendliness on one’ life satisfaction (Fig. 1).

## Methods

### Design

The study used a cross-sectional survey design.

### Population and sampling

According to Charan and Biswas [32], $\text{n}=\frac{{Z}_{1-\alpha /2}^{2}\times p(1-p)}{{d}^{2}}$, while ${Z}_{1-\alpha /2}$=1.96, *p*=0.409 (population aged 45 or above years accounted for 40.92% of the total population at the end of 2022) [33], *d*=0.05, the desired minimum sample size was 371. To achieve this, convenience and snowball sampling was implemented between August and September 2023. The inclusion criteria included Macao residents who (1) aged 45 years or above, (2) could speak Cantonese or Mandarin, and (3) were able to give consent and agreed to participate in the study. Those who did not understand the content of the questionnaire were excluded.

### Data collection and instruments

An online structured questionnaire was disseminated via advertisement and social media, as well as various local social services for older people. An introduction to the study was included at the beginning of the online questionnaire. Participants were asked to read the information, confirmed that they meet the criteria mentioned above, and select “Yes”. On the same page as the consent page, a question appeared that asked “I have clearly understood the above content”, participants to select “Consent” and continue to questionnaire.

The questionnaire consisted of four sections, including participant’s demographic information, Age-Friendly City (AFC) Checklist, Attitudes to Ageing Questionnaire (AAQ), Satisfaction with Life Scale (SWLS). All measurement tools were approved by the original authors for the study to use. The questionnaire was reviewed by three experts in the field of health and social care, including a nurse, a doctor, and a social worker. The scale-level Content Validity Index were 0.97 to 1.0. The questionnaire was considered suitable for the purpose of the study. In order to test the applicability of the questionnaire, a pilot was implemented on the targeted population with 30 participants. There was no change needed after analyzing the result from the pilot study.

#### Demographic Information

This section included participant’s gender, age, education level, marital status, whether they had children, living status, employment status, and self-rated health (SRH).

#### Age-friendliness

Age-Friendly City (AFC) Checklist developed by WHO were used to measure participants’ perception of age-friendliness. It was designed for older people residing in cities. This study adapted Chinese version of AFC criteria that has been used in Hong Kong [34]. The criteria involved 53 items under 8 domains. Participants rated their agreement with the AFC items using a 6-point Likert scale, ranging from 1 (strongly disagree) to 6 (strongly agree), the total score with higher scores indicating greater age-friendliness. In the current study, the overall Cronbach’s alpha of the AFC Checklist was 0.986 (0.988 for participants aged 45 to 64, and 0.982 for participants aged 65 and over), the eight subscales were between 0.89 and 0.95.

#### Attitudes to ageing

Attitudes to Ageing Questionnaire (AAQ) was used to assess participants’ attitudes to the process of ageing. AAQ was developed by Laidlaw et al. [35] using older participants across 20 countries. It consists of three subscales and 24 items, including psychosocial loss, psychological growth, and physical change. Each subscales contains 8 items. Participants rated the statements from 1 (strongly disagree) to 5 (strongly agree). The total score of each subscale ranges from 8 to 40. With higher scores in psychosocial loss subscale indicating more negative change in psychosocial aspect of ageing. While higher scores in psychological growth and physical change subscales indicating more positive change. This study used the Chinese version of AAQ, which was validated by Huang et al. [36]. The Cronbach’s alpha of psychosocial loss, psychological growth, and physical change subscales in this study were 0.868, 0.800, and 0.835 respectively.

#### Life satisfaction

Satisfaction with Life Scale (SWLS) was employed to evaluate participants’ global life satisfaction [37]. Participants were asked to rate the 5 statements using a 7-point Likert scale, ranging from 1 (strongly disagree) to 7 (strongly agree). The total score ranged from 5 to 35, with higher scores indicating greater satisfaction. This study adopted the Chinese version of SWLS that was translated by Xiong and Xu [38]. The Cronbach’s alpha in this study was 0.911.

### Data analysis

There was no missing data in the dataset. The data were summarized using descriptive statistics such as mean, standard deviation, frequency, and percentage. T-test and ANOVA were used to examine the correlation between participants characteristics and life satisfaction. The correlation between AFC, AAQ, and SWLS were examined by using Pearson correlation.

The proposed model was evaluated for its fitness using structural equation modeling. The model’s goodness of fit was evaluated through the maximum likelihood estimation method. The adjustment of the model was evaluated using the following model-fit indexes: root mean square error of approximation (RMSEA) ≤ 0.08 [39]; comparative fit index (CFI) ≥ 0.9, goodness of fit index (GFI), and Tucker-Lewis index (TLI) [40]. The modified model’s direct, indirect, and total effects were tested using the bootstrap method 2000 times within a 95% confidence interval. SPSS version 24 was used in descriptive, correlation, and multiple linear regression statistical procedures, while Amos version 23 was used to examine the mediating effect. A significant level of 5% was adopted.

## Results

The study received a total of 543 valid responses, with the majority of participants were female (64.83%). Middle-aged adults constituted a significant proportion of the participants (64.64%). Most of them hold an education level of secondary school or above (81.77%), were cohabited/ married (78.27%). had children (83.24%), with living with others (86.74%), and rated their health as fair or bad (63.35%). There are significant differences between age groups and individuals who their health as good and fair or bad. No differences observed in other characteristics (Table 1).

**Table 1:** Correlation between socio-demographic characteristics of the participants and SWLS (*n* = 543)

Variables		*n*	%	Mean	SD	F/ t	*p*
Gender						-0.07	0.94
	Male	191	35.17	24.04	5.80		
	Female	352	64.83	24.07	5.60		
Age (years)						-5.67	< 0.001
	45 to 64	351	64.64	23.03	5.19		
	≧ 65	192	35.36	25.95	6.01		
Education level						3.46	0.03^a^
	Primary school or below	99	18.23	25.34	7.53		
	Secondary school	225	41.44	23.56	5.33		
	Associate degree or above	219	40.33	24.00	4.91		
Marital status							
	Never married	40	7.37	23.13	4.42	0.94	0.39
	Cohabited/ married	425	78.27	24.23	5.62		
	Separated/ Divorced/ Widowed	78	14.36	23.64	6.41		
Children							
	None	91	16.76	23.64	4.76		
	Yes	452	83.24	24.15	5.83		
Living status						-0.32	0.75
	Living alone	72	13.26	23.86	6.37		
	Living with others	471	86.74	24.09	5.56		
Self-rated health						-6.79	< 0.001
	Fair/ (very) bad	344	63.35	22.85	5.70		
	(Very) Good	199	36.65	26.15	4.96		

^a^ Homogeneity of variance was not assumed, Games-Howell method was used as post-hoc comparison

**p < 0.01, ***p < 0.001

SD = standard deviation

**Fig. 2:**
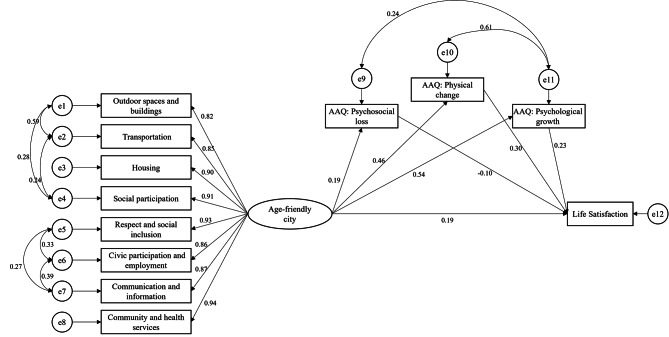
Model of the mediating effect of attitudes to ageing

The average score of AFC was 4.25, the score of psychosocial loss, physical change, and psychological growth of AAQ were 24.06, 29.00, and 26.94 respectively. The score of SWLS was 24.06. There was no significant correlation between psychosocial loss and life satisfaction (Table 2). After controlling age, education level, and self-rated health, AFC had significant direct effect (*β* = 0.187, *p* = 0.001, 95% CI [0.107, 0.266]), indirect effect through attitudes to ageing (*β* = 0.239, *p* = 0.001, 95% CI [0.184, 0.300]), and total effect (*β* = 0.426, *p* = 0.001, 95% CI [0.355, 0.501]) on life satisfaction. The model fit showed an adequate adjusted model (*X*^2^(*df*) = 137.447(64), *p* < 0.001; RMSEA = 0.046; CFI = 0.989; GFI = 0.966; TLI = 0.982). Suggesting that there was a partial mediation of attitudes to ageing in the relationship of perception of age-friendly city and life satisfaction (Fig. 2). The mediation effect explained 56.10% of the total effect of AFC to life satisfaction. A model 2, a significant variation was observed for participants ages 65 and over, the indirect effect of physical change in attitudes to ageing was not observed. Also, the mediation effect only explained 37.80% of the total effect of AFC to life satisfaction (Table 3).

**Table 2:** Correlation between AFC, AAQ, and SWLS (*n* = 543)

	SWLS	AFC mean	AFC (A)	AFC (B)	AFC (C)	AFC (D)	AFC (E)	AFC (F)	AFC (G)	AFC (H)	AAQ (PL)	AAQ (PC)	AAQ (PG)
SWLS	1												
AFC mean	0.444***	1											
AFC (A): Outdoor spaces and buildings	0.381***	0.903***	1										
AFC (B): Transportation	0.379***	0.925***	0.876***	1									
AFC (C): Housing	0.440***	0.896***	0.774***	0.782***	1								
AFC (D): Social participation	0.400***	0.925***	0.816***	0.826***	0.821***	1							
AFC (E): Respect and social inclusion	0.455***	0.918***	0.744***	0.775***	0.834***	0.846***	1						
AFC (F): Civic participation and employment	0.374***	0.867***	0.694***	0.717***	0.778***	0.766***	0.859***	1					
AFC (G): Communication and information	0.392***	0.888***	0.725***	0.749***	0.760***	0.789***	0.854***	0.845***	1				
AFC (H): Community and health services	0.439***	0.923***	0.766***	0.797***	0.844***	0.855***	0.867***	0.812***	0.825***	1			
AAQ (PL): psychosocial loss	0.037	0.196***	0.178***	0.191***	0.222***	0.172***	0.175***	0.161***	0.162***	0.157***	1		
AAQ (PC): physical change	0.609***	0.474***	0.398***	0.386***	0.414***	0.430***	0.487***	0.457***	0.469***	0.463***	0.109*	1	
AAQ (PG): psychological growth	0.553***	0.537***	0.456***	0.468***	0.479***	0.469***	0.546***	0.529***	0.494***	0.508***	0.330***	0.717***	1

*p < 0.05, ***p < 0.001

**Table 3:** Standardized effects (*β*) of mediation of attitudes to ageing to life satisfaction

Type	Effect	Model 1: Full sample (*n* = 543)					Model 2: Participants aged 65 and over (*n* = 192)			
		Estimate	95% C.I.		*p*		Estimate	95% C.I.		*p*
			Lower	Upper				Lower	Upper	
Indirect	AFC > SWLS	0.239	0.184	0.300	0.001		0.206	0.081	0.335	0.002
	AFC > AAQ_PL > SWLS	-0.020	-0.016	-0.008	0.005		-0.077	-0.058	-0.059	< 0.001
	AFC > AAQ_PC > SWLS	0.137	0.076	0.207	0.001		0.101	-0.003	0.254	0.056
	AFC > AAQ_PG > SWLS	0.123	0.061	0.198	0.001		0.182	0.030	0.373	0.009
Direct	AFC > SWLS	0.187	0.107	0.266	0.001		0.338	0.185	0.510	0.001
	AFC > AAQ_PL	0.194	0.090	0.297	0.001		0.319	0.157	0.465	0.001
	AAQ_PL > SWLS	-0.103	-0.174	-0.027	0.005		-0.241	-0.368	-0.127	0.001
	AFC > AAQ_PC	0.456	0.376	0.527	0.001		0.573	0.455	0.668	0.001
	AAQ_PC > SWLS	0.301	0.201	0.393	0.001		0.176	-0.007	0.380	0.061
	AFC > AAQ_PG	0.540	0.466	0.612	0.001		0.665	0.557	0.759	0.001
	AAQ_PG > SWLS	0.227	0.130	0.323	0.001		0.274	0.054	0.491	0.012
Total	AFC > SWLS	0.426	0.355	0.501	0.001		0.545	0.422	0.649	0.001

Model 1 controlled for age, education level, and self-rated health

Model 2 controlled for education level, and self-rated health

## Discussion

Life satisfaction is a universal assessment of one’s life based on one’s subjective criteria. It can reflect an individual’s life adaptation according to their internal expectations [37]. This study demonstrates the relationship of middle-aged and older people’s perception of the friendliness of a city and their life satisfaction, and the effect of their attitudes to ageing in between.

Based on the results of this study, the mean AFC score was 4.25, which is higher than the score of 3.99 obtained in five districts in Hong Kong [41]. Suggesting people in Macao perceived more age-friendliness. Macao is a small city with total land area of 33 km^2^ and total population of 672,800 [42]. Despite the projection suggesting that small cities are at higher risk of old-age dependency [43], Macao SAR government launched ageing-in-place policy in 2016 [44], preparing to response the rapid old-age dependency and to support its ageing populations, which might lead to the rather satisfactory age-friendliness among people in Macao.

In terms of attitudes to ageing, the average scores of psychosocial loss, physical change, and psychological growth subscales were 24.06, 29.00, and 26.94 respectively in the current study. Compared to a study conducted in the United Kingdom [46], the results in the current study had less negative perception of psychosocial loss, as well as less positive perception of physical change and psychological growth. One possible explanation is that the participants in this study were considerably younger. Participants in the UK study aged 70 and over accounted for 67.9%. However, participants aged 65 and over only accounted for only 35.36% in our study. It appears that there is an age difference in regard to one’s attitudes to ageing. Previous studies have been demonstrated that negative self-perceptions of ageing are closely associated with psychological well-being and quality of life of older people [47, 48]. There is a less polarization in the attitudes to ageing among middle-aged and older people in Macao, but it is still important to pay attention to the long-term impact of attitudes to ageing on their health.

This study found that the total score of SWLS was 24.06, there were significant differences between age and SRH groups. The score is higher than Japan with a score of 21.6 in younger age group and 22.5 in older age group [49], Hong Kong with a score of 23.35 in participants who aged 55 years and above [12], and older Chinese people living in rural areas with a score of 22.4 [50]. The result of people who aged 65 or above had higher score of SWLS is similar to the findings of previous studies. Studies in Taiwan [51] and Portugal [52] found that age and life satisfaction had a U-shaped relationship. The reason for this may be that older adults had more positive and stable emotion, as well as seeing old age is a blessing and is time to enjoy life, while more life stress and economic burden in midlife [53, 54]. People who rated their health as good or very good were more satisfied with their lives, which is similar to previous results [50, 55].

This study demonstrates the mediation role of one’s perception of their own ageing in the relationship of AFC and life satisfaction. According to Laidlaw et al. [35], psychosocial loss is individuals who view old age as a negative experience resulting in psychological and social decline. Physical change is perspective on the physical aspects of ageing and their relationship to health behaviors. Psychological growth is seeing positive gains in relation to self and others as you age. Such individual experiences and perspectives on ageing may be influenced by their society’s view of ageing. Stereotype embodiment theory argues that age stereotype can continue to function in individuals because individuals live in society through three possible pathways: physiological, behavioral, and psychological [56]. The effect of stereotype operates on individuals over time through implicit and explicit social interactions and internalization, which could not be reversed through short-term intervention. An experiment conducted by Fung et al. [57] showed that middle-aged participants did not react differently before and after positive portrayals of old age. Suggesting that the importance of long-term development of a positive view on ageing, which is what an age-friendly city can provide. On the other hand, person–environment fit theory suggests that individual’s adaptability to ageing varies with environmental support [58]. In an environment that encourages rather than constrains, a person would have a greater opportunity to engage in daily activities. Integrating these two theories, through the promotion of facilities construction and social interaction, AFC alters the public’s perception about ageing, thereby improving individual’s life satisfaction. Further analysis indicated that perception of age-friendliness positively influenced physical change, but that physical change did not influence life satisfaction of older people. Suggesting that for older population in Macao, increasing the perception of age-friendliness can improve their psychological status, thereby improving life satisfaction. As a study in Hong Kong found that age-friendly cities can lead to a greater sense of community, which in turn improves life satisfaction [12].

The total effect of AFC on SWLS was 42.6%. The effect of AFC on SWLS was greater than the result of Hong Kong, which AFC was only accounted for 14–15% of SWLS variance [17]. In addition, the result that the mediation effect accounted for 56.1% of the total impact of AFC on life satisfaction enriches the understanding of how people’s perception of age-friendliness impacts their life satisfaction. One thing to note is that this article examines the influence of age-friendly cities on ageing attitudes from the perspective that social construction will affect individual attitudes to ageing. Future research is recommended to examine whether attitudes toward ageing can influence perceptions of age-friendliness as well.

### Study limitations

There were a few limitations to the current study. First, the representativeness of sample population as it adopted the convenient and snowball sampling methods. Therefore, generalization of the study’s results might be limited. The results may only reflect the older people who are easily accessed. Those with limited access to community may not be included in the results. Also, convenience and snowball sampling may be more susceptible to social desirability bias. Those with certain characteristics or affiliations are likely to refer others with similar views or opinions, resulting in potentially biased results. Second, there was no causal relationships between variables determined due to the cross-sectional nature of the data. Further studies are needed to clarify the causal relationships between variables.

## Conclusions

This study demonstrates that perception of age-friendliness of a city is associated with life satisfaction, and the effect of perception of age-friendliness on life satisfaction is partially mediated by attitudes to ageing. The development of an age-friendly city can help improve the public’s view on ageing by demonstrating that older people are welcomed by society and can live independently as they adapt to the process of ageing, thus improve their life satisfaction. It is important for government to consider the improvement of people’s attitudes to ageing when developing policies regarding AFC, not just to support the current ageing population, but for the future generations as well.

## Data Availability

The datasets used and/or analyzed during the current study available from the corresponding author on reasonable request.

## Abbreviations

AFC: Age-friendly city
AAQ: Attitudes to Ageing Questionnaire
SWLS: Satisfaction with Life Scale

## References

[1] Statistics and Census Service of Government of Macao Special Administrative Region. Projecções da População de Macau 2022–2041. 2023a.

[2] Statistics and Census Service of Government of Macao Special Administrative Region. Demographic Statistics 2022. 2023b.

[3] Cristea M, Noja GG, Stefea P, Sala AL (2020). The Impact of Population Aging and Public Health support on EU Labor Markets. Int J Environ Res Public Health.

[4] Rogers WA, Ramadhani WA, Harris MT. Defining aging in place: the intersectionality of space, person, and time. Innov Aging. 2020;4(4). 10.1093/geroni/igaa036 PMC7595274 33173834

[5] World Health Organization. Global Age-Friendly Cities: A Guide 2007 [ https://www.who.int/publications/i/item/9789241547307

[6] The Government of the Macao Special Administrative Region. Mecanismo De Protecção dos Idosos E Plano Decenal De Acção para os Serviços De Apoio a Idosos 2016–2025 Da Região. Administrativa Especial de Macau; 2016.

[7] Lei n.º 12/2018 Regime jurídico de garantias dos direitos e interesses dos idosos. (2018).

[8] Ng S-I, Lim X-J, Hsu H-C (2021). The importance of Age-Friendly City on older people’s continuity and life satisfaction. Int J Environ Res Public Health.

[9] Zhou J-J, Kang R, Bai X. A meta-analysis on the influence of age-friendly environments on older adults’ physical and mental well-being. Int J Env Res Pub He. 2022;19(21):13813. 10.3390/ijerph192113813 PMC9657613 36360692

[10] Lu N, Wu B (2022). Perceived neighborhood environment, social capital and life satisfaction among older adults in Shanghai, China. Sci Rep.

[11] Chung S, Kim M (2023). Age-friendly environment, social support, sense of community, and loneliness among middle-aged and older adults in Korea. Aging Ment Health.

[12] Au A, Lai DWL, Yip H-m, Chan S, Lai S, Chaudhury H et al. Sense of community mediating between age-friendly characteristics and life satisfaction of community-dwelling older adults. Front Psychol. 2020;11. 10.3389/fpsyg.2020.00086 PMC7064721 32194465

[13] Zamora FMV, Kloseck M, Fitzsimmons DA, Zecevic A, Fleming P (2020). Use of community support and health services in an age-friendly city: the lived experiences of the oldest-old. Cities Health.

[14] Flores R, Caballer A, Alarcón A (2019). Evaluation of an age-friendly City and its effect on life satisfaction: a two-stage study. Int J Environ Res Public Health.

[15] Pan Z, Liu Y, Liu Y, Huo Z, Han W (2024). Age-friendly neighbourhood environment, functional abilities and life satisfaction: a longitudinal analysis of older adults in urban China. Soc Sci Med.

[16] Xie L (2018). Age-Friendly communities and life satisfaction among the Elderly in Urban China. Res Aging.

[17] Au AML, Chan SCY, Yip HM, Kwok JYC, Lai KY, Leung KM (2017). Age-friendliness and life satisfaction of Young-Old and Old-Old in Hong Kong. Curr Gerontol Geriatr Res.

[18] Makita M, Mas-Bleda A, Stuart E, Thelwall M (2021). Ageing, old age and older adults: a social media analysis of dominant topics and discourses. Aging Soc.

[19] Mapoma CC, Masaiti G (2012). Perceptions of and attitudes towards Ageing in Zambia. Eur J Educational Res.

[20] Wurm S, Diehl M, Kornadt AE, Westerhof GJ, Wahl HW (2017). How do views on aging affect health outcomes in adulthood and late life? Explanations for an established connection. Dev Rev.

[21] Thorpe AM. Attitudes to ageing: relationships with health and health behaviours at midlife. University of Otago; 2014.

[22] Singh DKA, Ibrahim A, Chong PK, Subramaniam P (2018). Attitude towards ageing and physical performance among adults 55 years old and above. Malaysian J Public Health Med.

[23] Tully-Wilson C, Bojack R, Millear PM, Stallman HM, Allen A, Mason J (2021). Self-perceptions of aging: a systematic review of longitudinal studies. Psychol Aging.

[24] Lamont RA, Swift HJ, Abrams D (2015). A review and meta-analysis of age-based stereotype threat: negative stereotypes, not facts, do the damage. Psychol Aging.

[25] Bodner E, Ayalon L, Avidor S, Palgi Y (2017). Accelerated increase and relative decrease in subjective age and changes in attitudes toward own aging over a 4-year period: results from the Health and Retirement Study. Eur J Ageing.

[26] Warmoth K, Tarrant M, Abraham C, Lang IA (2016). Older adults’ perceptions of ageing and their health and functioning: a systematic review of observational studies. Psychol Health Med.

[27] Westerhof GJ, Nehrkorn-Bailey AM, Tseng H-Y, Brothers A, Siebert JS, Wurm S (2023). Longitudinal effects of subjective aging on health and longevity: an updated meta-analysis. Psychol Aging.

[28] Levy BR, Slade MD, Kunkel SR, Kasl SV (2002). Longevity increased by positive self-perceptions of aging. J Personal Soc Psychol.

[29] Hajek A, König H-H (2020). Feeling too old? Consequences for subjective well-being. Longitudinal findings from the German ageing survey. Arch Gerontol Geriatr.

[30] Blöchl M, Nestler S, Weiss D (2021). A limit of the subjective age bias: feeling younger to a certain degree, but no more, is beneficial for life satisfaction. Psychol Aging.

[31] Suh S, Choi H, Lee C, Cha M, Jo I (2012). Association between knowledge and attitude about aging and life satisfaction among older koreans. Asian Nurs Res.

[32] Charan J, Biswas T (2013). How to calculate sample size for different study designs in Medical Research?. Indian J Psychol Med.

[33] Statistics and Census Service. Demographic Statistics 2022 2023 [ https://www.dsec.gov.mo/en-US/Statistic?id=101

[34] Wong M, Chau PH, Cheung F, Phillips DR, Woo J (2015). Comparing the age-friendliness of different neighbourhoods using district surveys: an Example from Hong Kong. PLoS ONE.

[35] Laidlaw K, Power MJ, Schmidt S (2007). The attitudes to Ageing Questionnaire (AAQ): development and psychometric properties. Int J Geriatr Psychiatry.

[36] Huang Y-f, Wang D-h, Liu Y-g (2010). Application of attitudes to Aging Questionnaire (AAQ) among Chinese aged adults. Chin J Clin Psychol.

[37] Diener E, Emmons RA, Larsen RJ, Griffin S (1985). The satisfaction with Life Scale. J Pers Assess.

[38] Xiong C, Xu Y (2009). Reliability and validity of the stisfaction with life scale for Chinese demos. China J Health Psychol.

[39] Browne MW, Cudeck R (1992). Alternative ways of assessing Model Fit. Sociol Methods Res.

[40] Musil CM, Jones SL, Warner CD (1998). Structural equation modeling and its relationship to multiple regression and factor analysis. Res Nurs Health.

[41] Yu R, Wong M, Woo J (2019). Perceptions of Neighborhood Environment, Sense of Community, and self-rated health: an age-friendly City Project in Hong Kong. J Urb Health.

[42] Government Information Bureau of the Macao Special Administrative Region. Macao Yearbook 2023. 2023.

[43] Hartt MD, Biglieri S (2018). Prepared for the silver tsunami? An examination of municipal old-age dependency and age-friendly policy in Ontario, Canada. J Urban Affairs.

[44] Government of Macao Special Administrative Region. Mecanismo De Protecção dos Idosos E Plano Decenal De Acção para os Serviços De Apoio a Idosos 2016–2025 Da Região. Administrativa Especial de Macau; 2016.

[46] Thelu M, Webster B, Jones K, Orrell M (2022). A cross sectional survey on UK older adult’s attitudes to ageing, dementia and positive psychology attributes. BMC Geriatr.

[47] Losada-Baltar A, Jiménez-Gonzalo L, Gallego-Alberto L, Pedroso-Chaparro MdS, Fernandes-Pires J, Márquez-González M (2020). We are staying at Home. Association of Self-perceptions of Aging, Personal and Family resources, and loneliness with psychological distress during the lock-down period of COVID-19. Journals Gerontology: Ser B.

[48] Joshanloo M. Longitudinal relationship between self-perceptions of aging and depressive symptoms: exploring reciprocal within-person links. Int J Mental Health Addict. 2023.

[49] Kida H, Niimura H, Eguchi Y, Suzuki K, Shikimoto R, Bun S, et al. Relationship between life satisfaction and psychological characteristics among community-dwelling oldest-old: focusing on Erikson’s developmental stages and the big five personality traits. Am J Geriat Psychiat. 2023. 10.1016/j.jagp.2023.12.018 38216354

[50] Xiao Q, Wang Y, Li J, Li J. Relationship between social support and life satisfaction among the rural elderly: mediating effect and moderating effect. Chin Mental Health J. 2018;32(2).

[51] An H-Y, Chen W, Wang C-W, Yang H-F, Huang W-T, Fan S-Y (2020). The relationships between physical activity and life satisfaction and happiness among Young, Middle-Aged, and older adults. Int J Environ Res Public Health.

[52] Tavares AI (2022). Health and life satisfaction factors of Portuguese older adults. Arch Gerontol Geriatr.

[53] Chen C (2001). Aging and life satisfaction. Soc Indic Res.

[54] Carstensen LL, Turan B, Scheibe S, Ram N, Ersner-Hershfield H, Samanez-Larkin GR (2011). Emotional experience improves with age: evidence based on over 10 years of experience sampling. Psychol Aging.

[55] Rajabi Gilan N, khezeli M, Zardoshtian S (2021). The effect of self-rated health, subjective socioeconomic status, social capital, and physical activity on life satisfaction: a cross-sectional study in urban western Iran. BMC Public Health.

[56] Levy B (2009). Stereotype embodiment: a Psychosocial Approach to Aging. Curr Dir Psychol Sci.

[57] Fung HH, Li T, Zhang X, Sit IM, Cheng ST, Isaacowitz DM (2015). Positive portrayals of Old Age do not always have positive consequences. J Gerontol B Psychol Sci Soc Sci.

[58] Lawton MP, Nahemow L (1973). Ecology and the aging process. The psychology of adult development and aging.

